# Generalized Haldane models on laser-coupling optical lattices

**DOI:** 10.1038/s41598-018-30503-9

**Published:** 2018-08-27

**Authors:** Wanli Liu, Zhi Lin, Z. D. Wang, Yan Chen

**Affiliations:** 1 Department of Physics and State Key Laboratory of Surface Physics, Fudan University, Shanghai, 200438 China; 2 Shenzhen Institute of Research and Innovation, The University of Hong Kong, Shenzhen, 518063 P.R. China; 3 Department of Physics and Center of Theoretical and Computational Physics, The University of Hong Kong, Pokfulam Road, Hong Kong, China; 4 Collaborative Innovation Center of Advanced Microstructures, Nanjing, 210093 China

## Abstract

We propose two generalized Haldane models on laser-coupling optical lattices. Laser-assisted nearest neighbour tunnelings generate artificial staggered magnetic flux, facilitating the realization of topological nontrivial band structures. As generalizations of Haldane model, these models support topological insulator and semimetal phases featuring high Chern numbers. We show simple rules for computing Chern numbers of our models and display the phase diagrams. Moreover, numerical calculations of energy spectra are in perfect agreement with our theoretical expectations. Our models may serve as two new family members for generalizing Haldane model on optical lattices.

## Introduction

Topologically ordered phases of matter have been a hot topic in condensed matter physics since the discovery of the integer quantum Hall effect^1^. In lattice systems, the topologically nontrivial band structures are currently attracting a great deal of interest^2,3^. The band topology is characterized by a topological invariant-Chern number-taking^4^ integer values and signaling topological order. Nontrivial topological order has experimental consequences, such as quantized Hall conductivity^5^ and the existence of edge states^2,3^. In two-dimensional lattice systems, one of the simplest models supporting topological bands was proposed by Haldane^6^. This model features real nearest-neighbor (NN) hopping and complex next-nearest-neighbor (NNN) hopping on a honeycomb lattice. Haldane showed that this two-band model is topologically equivalent to integer quantum Hall state.

In recent years, topological insulator^7–10^, topological superconductor^11–13^ and topological semimetal^14–19^ have been widely explored. To simulate these fascinating quantum phenomena, ultracold atoms in optical lattice is an ideal playground, for its versatile control of interactions which are hard to adjust in traditional solid state systems^20^. Several methods have been proposed to realize topological nontrivial band structures with cold atoms, such as lattice shaking^21–23^, rotation^24^, laser coupling^25–28^, or using synthetic dimensionality^29^. Recently, Haldane model has been realized experimentally through lattice shaking^30^. Measurement of Chern number of Hofstadter bands has been successfully performed^31^. The quantized conductance in neutral matter system has also been observed^32^. Besides, there are many other developments on Chern number detection^33,34^, models design that support nontrivial topology^35–37^ and so on. Recently, some three-band tight-binding models have been proposed^38–40^, because of their high Chern numbers and easy access to topological semimetal phases.

In the present paper, we propose two simple lattice models that support topological insulator and semimetal phases featuring high Chern numbers. Our tight-binding model is defined on laser-coupling optical lattice, on which complex NN tunnelings are laser assisted. The three-band model is defined on state-dependent lattice whereas the four-band model is on state-independent lattice, both pierced by effective staggered magnetic fields. As generalizations of Haldane model, these models support topological nontrivial energy bands. Chern numbers are computed in a simple way and rich phase diagrams are exhibited. Moreover, the topological edge states show time-reversal (TR) symmetry for a special case, although TR symmetry breaking in the bulk is required for a nonzero Chern number.

## Results

### Three-band Model

**Figure 1:**
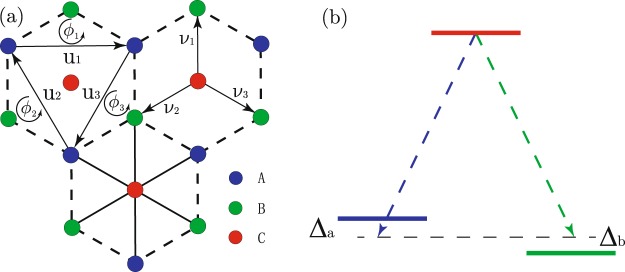
(**a**) Honeycomb-like lattice with NN vectors *ν*_*i*_ and NNN vectors **u**_**i**_, pierced by effective magnetic flux ${\varphi }_{i}={\bf{p}}\cdot {{\bf{u}}}_{{\bf{i}}}/\mathrm{2\; (}i=1,2,3)$. Atoms of three different states are trapped on inequivalent sites. (**b**) State A and B are coupled with state C by two laser beams, with detuning Δ_*a*_ and Δ_*b*_.

In our three-band model, fermionic ultracold atoms are trapped on honeycomb-like lattice. Atoms of three different hyperfine states are located on inequivalent sites, labeled by A, B and C (Fig. 1a). In the tight-binding regime, Hamiltonian takes the form $\hat{H}={\hat{H}}_{T}+{\hat{H}}_{L}+{\hat{H}}_{V}$:

1 $$\begin{array}{rcl}{\hat{H}}_{T} & = & -t\sum _{\langle \langle i,j\rangle \rangle }({\hat{a}}_{i}^{\dagger }{\hat{a}}_{j}+{\hat{b}}_{i}^{\dagger }{\hat{b}}_{j}+{\hat{c}}_{i}^{\dagger }{\hat{c}}_{j})\\ {\hat{H}}_{{\rm{L}}} & = & {\lambda }_{a}\sum _{\langle i,j\rangle }{\hat{c}}_{i}^{\dagger }{\hat{a}}_{j}{e}^{i{\varphi }_{ij}}+{\lambda }_{b}\sum _{\langle i,j\rangle }{\hat{c}}_{i}^{\dagger }{\hat{b}}_{j}{e}^{i{\varphi }_{ij}}+h\,.\,c.\\ {\hat{H}}_{V} & = & {{\rm{\Delta }}}_{a}\sum _{j}{\hat{a}}_{j}^{\dagger }{\hat{a}}_{j}+{{\rm{\Delta }}}_{b}\sum _{j}{\hat{b}}_{j}^{\dagger }{\hat{b}}_{j}.\end{array}$$

where ${\hat{a}}_{i},{\hat{b}}_{i},{\hat{c}}_{i}$ correspond to annihilation operators on site *i* of three sublattices. Normal NNN tunnelings of amplitude *t* take place within the same triangular sublattices. Laser-assisted NN tunnelings^41–43^ of amplitude *λ*_*a*(*b*)_ are accompanied by recoil momentum **p** (Fig. 1b), generating the Peierls phases^41^ ${\varphi }_{ij}={\bf{p}}\cdot ({{\bf{r}}}_{i}+{{\bf{r}}}_{j})/2$. For simplicity, we assume recoil momentums **p** of the two coupling processes are the same. Within the framework of the rotating-wave approximation, resonance detunings Δ_*a*(*b*)_ of the laser coupling processes behave like onsite potentials. To eliminate the explicit spatial dependence of our Hamiltonian, we perform a unitary transformation^36^

2 $${\hat{a}}_{j}\to {\hat{a}}_{j}{e}^{-i{\bf{p}}\cdot {{\bf{r}}}_{j}/2},\,{\hat{b}}_{j}\to {\hat{b}}_{j}{e}^{-i{\bf{p}}\cdot {{\bf{r}}}_{j}\mathrm{/2}},\,{\hat{c}}_{j}\to {\hat{c}}_{j}{e}^{i{\bf{p}}\cdot {{\bf{r}}}_{j}\mathrm{/2}}.$$

Thus giving a transformed Hamiltonian of real NN tunnelings and complex NNN tunnelings

3 $$\begin{array}{rcl}{\hat{H}}_{T} & = & -t\sum _{j,{u}_{i}}({\hat{a}}_{j}^{\dagger }{\hat{a}}_{j+{u}_{i}}{e}^{-i{\varphi }_{i}}+{\hat{b}}_{j}^{\dagger }{\hat{b}}_{j+{u}_{i}}{e}^{-i{\varphi }_{i}}+{\hat{c}}_{j}^{\dagger }{\hat{c}}_{j+{u}_{i}}{e}^{i{\varphi }_{i}}+h\mathrm{.}c\mathrm{.}),\\ {\hat{H}}_{L} & = & {\lambda }_{a}\sum _{\langle i,j\rangle }{\hat{c}}_{i}^{\dagger }{\hat{a}}_{j}+{\lambda }_{b}\sum _{\langle i,j\rangle }{\hat{c}}_{i}^{\dagger }{\hat{b}}_{j}+h.\,c.\,,\end{array}$$

where ${\varphi }_{i}={\bf{p}}\cdot {{\bf{u}}}_{i}\mathrm{/2}$ no longer depends on spatial coordinates, playing the role of effective staggered magnetic flux (Fig. 1a). The present Hamiltonian is invariant under discrete translation. Thus it can be written in momentum space $\hat{H}={\hat{{\rm{\Psi }}}}_{k}^{\dagger }{\hat{H}}_{k}{\hat{{\rm{\Psi }}}}_{k}$ with ${\hat{{\rm{\Psi }}}}_{k}={({\hat{a}}_{k},{\hat{c}}_{k},{\hat{b}}_{k})}^{T}$

4 $${\hat{H}}_{k}=(\begin{array}{ccc}{\xi }_{a}^{k} & {\lambda }_{a}^{k\ast } & 0\\ {\lambda }_{a}^{k} & {\xi }_{c}^{k} & {\lambda }_{b}^{k\ast }\\ 0 & {\lambda }_{b}^{k} & {\xi }_{b}^{k}\end{array}),$$

where

5 $$\begin{array}{rcl}{\xi }_{a(b)}^{k} & = & {{\rm{\Delta }}}_{a(b)}-\,2t\sum _{i}\cos ({\bf{k}}\cdot {{\bf{u}}}_{i}+{\varphi }_{i}),\\ {\xi }_{c}^{k} & = & -2t\sum _{i}\cos ({\bf{k}}\cdot {{\bf{u}}}_{i}-{\varphi }_{i}),\\ {\lambda }_{a(b)}^{k} & = & {\lambda }_{a(b)}\sum _{i}{e}^{{\rm{i}}{\bf{k}}\cdot {\nu }_{i}},\end{array}$$

**u**_**i**_(*i* = 1, 2, 3) is the NNN vector and *ν*_**i**_(*i* = 1, 2, 3) is the NN vector.

Given the momentum-space Hamiltonian (4), one easily knows there are three energy bands featured by Bloch wave functions *ψ*_*n*,*k*_ (*n* = 1, 2, 3). The topological order of each band is characterized by the Chern number

6 $${C}_{n}=\frac{1}{2\pi }{\int }_{BZ}{d}^{2}k{F}_{n}(k)=-\,\frac{1}{2\pi }\sum {\oint }_{singul}d{\bf{k}}\cdot {{\bf{A}}}_{n}$$

where ${F}_{n}(k)=i{\hat{e}}_{z}\cdot \langle {\overrightarrow{\nabla }}_{k}{\psi }_{n,k}|\times |{\overrightarrow{\nabla }}_{k}{\psi }_{n,k}\rangle$ is the Berry curvature and ${{\bf{A}}}_{n}(k)=i\langle {\psi }_{n,k}|{\overrightarrow{\nabla }}_{k}{\psi }_{n,k}\rangle$ is the Berry connection. Berry curvature can be expressed as the *z* component of curl ${F}_{n}(k)={\hat{e}}_{z}\cdot {\nabla }_{{\bf{k}}}\times {{\bf{A}}}_{{n}}(k)$. The Chern number is nonzero only if TR symmetry is breaking, or rather, it’s the artificial staggered magnetic flux ${\varphi }_{i}$ that gives rise to topological nontrivial band structures. Interestingly, the Chern numbers can be higher than one that is beyond the usual quantum Hall effect.

**Figure 2:**
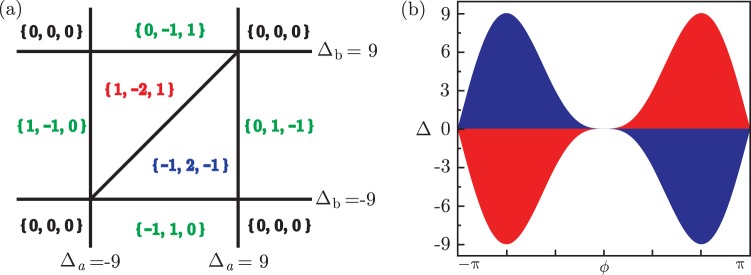
(**a**) Phase diagram of isotropic case ${\varphi }_{i}=2\pi \mathrm{/3}$. The Chern number of {up,middle,down} energy bands is labeled by {x, y, z}, respectively. (**b**) Topological nontrivial regions with boundaries $|{\rm{\Delta }}|=4\sqrt{3}|\,\sin \,\varphi (1-\,\cos \,\varphi )|$, red for {1, −2, 1} and blue for {−1, 2, −1}, where Δ = −Δ_*a*_ = Δ_*b*_ and ${\varphi }_{\mathrm{2,3}}=\varphi$.

We are more interested in two symmetric cases and show their phase diagrams. For the isotropic case ${\varphi }_{i}=2\pi /3$ shown in Fig. 2a, we identify different regions by Chern numbers {*x*, *y*, *z*} of three bands. Setting *t* as unit, high Chern number phases $\{\,\pm \,1,\,\mp \,2,\,\pm \,1\}$ emerge when $|{{\rm{\Delta }}}_{a(b)}| < 9$ whereas it’s trivial when $|{{\rm{\Delta }}}_{a(b)}| < 9$. If just one of Δ_*a*(*b*)_ becomes large, it reduces to an effective two-band model with Chern numbers $\mathrm{\{0,}\mp \,\mathrm{1,}\pm \,\mathrm{1\}}$ or $\{\,\mp \,\mathrm{1,}\pm \,\mathrm{1,}\,\mathrm{0\}}$. For the other symmetric case in Fig. 2b, parameters set as $-{{\rm{\Delta }}}_{a}={{\rm{\Delta }}}_{b}={\rm{\Delta }},{\varphi }_{\mathrm{2(3)}}=\varphi$, we have two topological nontrivial phases with high Chern numbers $\{\,\pm \,\mathrm{1,}\mp \,\mathrm{2,}\pm \,\mathrm{1\}}$. The condition of phase transition $({\xi }_{a}^{K}-{\xi }_{c}^{K})({\xi }_{b}^{K}-{\xi }_{c}^{K})=0$ gives rise to the phase boundary

7 $$|{\rm{\Delta }}|=4\sqrt{3}|\,\sin \,\varphi (1-\,\cos \,\varphi )|,$$

which deviates from the sine-shaped one of Haldane model by a factor $\mathrm{(1}-\,\cos \,\varphi )$. The magnetic flux *ϕ* affect the nontrivial scale of detuning Δ, allowing for the largest value |Δ| = 9 when it approaches ±2*π*/3. The coupling strength *λ*_*a*(*b*)_ has no influence on Chern numbers, but it turns out to modify the energy gaps and lead to topological semimetal phases as shown in the following numerical calculations.

**Figure 3:**
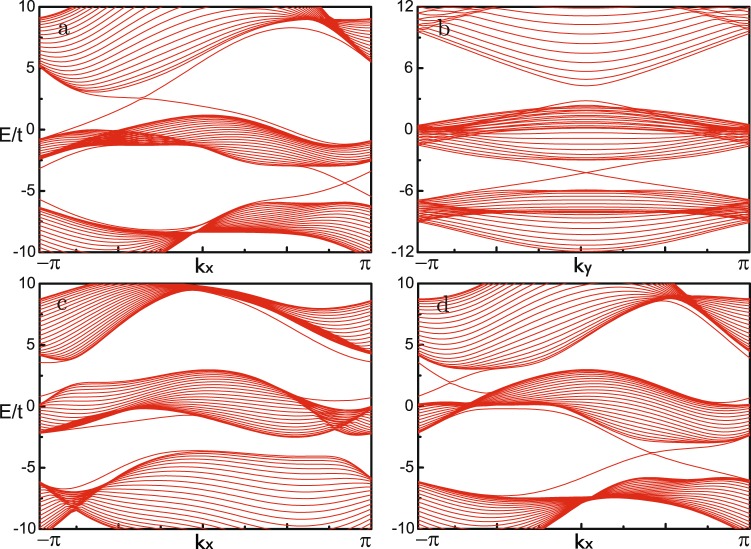
Energy spectra *E* as a function of quasi-momentum *k*. (**a**,**b**) Phase transition $\{\mathrm{1,}-\,2,1\}\to \{0,-\,\mathrm{1,}\,1\}$ driven by changes of detuning $({{\rm{\Delta }}}_{a},{{\rm{\Delta }}}_{b})=(\,-\,\mathrm{6,}\,6)\to (\,-\,6,\,10)$ when ${\lambda }_{a}=\mathrm{2,}\,{\lambda }_{b}=3$ and ${\varphi }_{i}=2\pi /3$. (**c**,**d**) Phase transition $\{\mathrm{0,}\,\mathrm{0,}\,0\}\to \{\,-\,\mathrm{1,}\,\mathrm{2,}-\,1\}$ driven by changes of magnetic flux ${\varphi }_{\mathrm{2(3)}}=\pi \mathrm{/3}\to 2\pi \mathrm{/3}$ when ${{\rm{\Delta }}}_{a}=-\,{{\rm{\Delta }}}_{b}=6$ and ${\lambda }_{a(b)}=2$.

The defining characteristic of topological nontrivial band structure is the existence of gapless edge states. We numerically calculate the energy spectra for 1D ribbons with zigzag and armchair edges, which is periodic in x and y direction respectively. The displayed edge states within the bulk energy gaps address different topological nontrivial phases. To demonstrate previous analytical results, we show two kinds of topological phase transitions driven by resonance detuning Δ or artificial magnetic flux *ϕ*. For the isotropic case of phase diagram Fig. 2a, topological phase transition $\{1,-\,2,1\}\to \{0,-\,1,1\}$ driven by detuning Δ is shown in Fig. 3a and b. One can see the edge states disappear between the upper two bands during the process of enlarging Δ_*b*_ with fixed Δ_*a*_. Specially, there is a symmetry $\hat{H}({k}_{x},{k}_{y})={\hat{H}}^{\ast }({k}_{x},-\,{k}_{y})$ for armchair case if ${\varphi }_{2}={\varphi }_{3}$ is maintained. Replacing *k*_*x*_ by −*i*∂_*x*_ and integrating over *x*, one can find effective edge Hamiltonian ${\hat{H}}_{edge}({k}_{y})={\hat{H}}_{edge}^{\ast }(\,-\,{k}_{y})$. So there emerges TR symmetry at the boundaries, resulting in a symmetric energy spectrum. For the other symmetric case of phase diagram Fig. 2b, topological phase transition $\mathrm{\{0,}\,\mathrm{0,}\,0\}\to \{-\,1,\mathrm{2,}-\,1\}$ driven by *ϕ* is presented in Fig. 3c and d. By tuning the artificial magnetic flux *ϕ*, two edge states show up between the two energy gaps. Here the upper gap is indirect zero, signaling topological semimetal phase. Comparing Fig. 3a and d, one knows that coupling strength *λ*_*a*(*b*)_ modifies the energy gap and results in topological insulator-semimetal phase transitions.

### Four-band model

In this section, we propose a similar model on honeycomb lattice by using internal states of ultracold atoms. The Hamiltonian takes the form *H* = *H*_*T*_ + *H*_*L*_ + *H*_*V*_:

8 $$\begin{array}{rcl}{\hat{H}}_{T} & = & -\,\sum _{\langle \langle i,j\rangle \rangle ,\alpha }{t}_{ij}{c}_{i\alpha }^{\dagger }{c}_{j\alpha }+h\mathrm{.}c\mathrm{.}\\ {\hat{H}}_{L} & = & \lambda \sum _{\langle i,j\rangle ,\alpha \beta }{c}_{i\alpha }^{\dagger }{\sigma }_{\alpha \beta }^{x}{c}_{j\beta }{e}^{i{\varphi }_{ij}}+h\mathrm{.}c.\\ {\hat{H}}_{V} & = & \sum _{j,\alpha \beta }{c}_{j\alpha }^{\dagger }(\varepsilon {\sigma }_{\alpha \beta }^{z}+d{\sigma }_{\alpha \beta }^{x}){c}_{j\beta }+{\delta }_{j}{c}_{j\alpha }^{\dagger }{c}_{j\alpha }\end{array}$$

**Figure 4:**
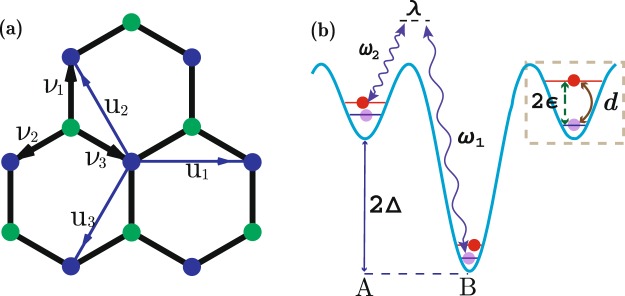
(**a**) Honeycomb optical lattice with NN vectors *ν*_*i*_ and NNN vectors *u*_*i*_(*i* = 1, 2, 3). (**b**) Atoms of different internal states with energy ±*ε*, show Rabi oscillation of strength *d*. NN tunnelling is laser-assisted through two-photon Raman process of strength *λ* with internal state changed, while the normal NN tunnelling is suppressed by the large potential barrier 2Δ between the two sublattices.

*H*_*T*_ is the NNN hopping term on the honeycomb lattice (Fig. 4a), where *c*_*jα*_ is the annihilation operator of fermion with pseudospin *α* (internal state $|e\rangle$ and $|g\rangle$) on site *j*. Tunneling strength ${t}_{ij}={t}_{a(b)}$ is different in two sublattices due to the trap potential depth difference 2Δ. *H*_*L*_ is laser-assisted NN tunneling via two-photon Raman process (Fig. 4b), and normal tunneling is suppressed by the large potential barrier 2Δ. We also introduce energy ±*ε* for internal states, dipole interaction *d* and staggered sublattice resonance detuning *δ*_*j*_ *=* ±*δ* in *H*_*V*_.

Atoms show dipole ${\bf{d}}=\wp |g\rangle \langle e|+\,{\wp }^{\ast }|e\rangle \langle g|$ interacting with electric field **E**, resulting in dipole interaction $-{\bf{d}}\cdot {\bf{E}}\sim d{\sigma }_{x}$. Two Raman laser beams facilitate the complex NN tunnelings of phase ${\varphi }_{ij}={\bf{p}}\cdot ({{\bf{r}}}_{i}+{{\bf{r}}}_{j})/2$ with transferred energy ${\omega }_{1}-{\omega }_{2}\approx 2{\rm{\Delta }}$ and momentum **p**, which has no relation with internal states but with the tunneling directions. Thus we have staggered sublattice resonance detuning $2\delta =\hslash ({\omega }_{1}-{\omega }_{2})-2{\rm{\Delta }}$ in rotating wave frame.

To eliminate the explicit spatial dependence of phase factor *ϕ*_*ij*_, unitary transformation ${\hat{c}}_{j}\to {\hat{c}}_{j}{e}^{\pm i{\bf{p}}\cdot {{\bf{r}}}_{j}/2}$ is performed, making the NN tunnelings real and NNN tunnelings complex. In the basis of ${{\rm{\Psi }}}_{k}={({\hat{a}}_{k\uparrow },{\hat{a}}_{k\downarrow },{\hat{b}}_{k\uparrow },{\hat{b}}_{k\downarrow })}^{T}$, momentum Hamiltonian $H={{\rm{\Psi }}}_{k}^{\dagger }{H}_{k}{{\rm{\Psi }}}_{k}$ is written as

9 $${\hat{H}}_{k}=(\begin{array}{cccc}{t}_{k}^{a}-\delta +\varepsilon  & d & 0 & {\lambda }_{k}\\ d & {t}_{k}^{a}-\delta -\varepsilon  & {\lambda }_{k} & 0\\ 0 & {\lambda }_{k}^{\ast } & {t}_{k}^{b}+\delta +\varepsilon  & d\\ {\lambda }_{k}^{\ast } & 0 & d & {t}_{k}^{b}+\delta -\varepsilon \end{array}),$$

where ${t}_{k}^{a,b}=-\,2{t}_{a,b}{\sum }_{i}\,\cos ({\bf{k}}\cdot {{\bf{u}}}_{i}\pm {\varphi }_{i}),\,{\lambda }_{k}=\lambda {\sum }_{i}{e}^{i{\bf{k}}\cdot {\nu }_{{\bf{i}}}}$ with phases ${\varphi }_{i}={\bf{p}}\cdot {{\bf{u}}}_{i}/2$. *H*_*k*_ can be expanded as

10 $$\begin{array}{rcl}{\hat{H}}_{k} & = & {t}_{k}^{0}I+({t}_{k}^{1}-\delta )I\otimes {s}_{z}+\varepsilon {\sigma }_{z}\otimes I+d{\sigma }_{x}\otimes I\\  &  & +Re({\lambda }_{k}){\sigma }_{x}\otimes {s}_{x}-Im({\lambda }_{k}){\sigma }_{x}\otimes {s}_{y},\end{array}$$

where ${t}_{k}^{\mathrm{0,1}}=({t}_{k}^{a}\pm {t}_{k}^{b})\mathrm{/2}$ and Pauli matrices *σ*_*i*_ and *s*_*i*_ representing the spin and sublattice indices. There are four energy bands

11 $${E}_{k}={t}_{k}^{0}\pm \sqrt{{d}^{2}+{\varepsilon }^{2}+|{\lambda }_{k}{|}^{2}+{({t}_{k}^{1}-\delta )}^{2}\pm 2\sqrt{({d}^{2}+{\varepsilon }^{2}){({t}_{k}^{1}-\delta )}^{2}+{d}^{2}|{\lambda }_{k}{|}^{2}}}$$

and the two middle bands touch at Dirac points $(K,K^{\prime} )$ when ${({t}_{k}^{1}-\delta )}^{2}={\varepsilon }^{2}+{d}^{2}$. It implies that half filled system is topological nontrivial when $\delta \in ({t}_{K}^{1}-\sqrt{{\varepsilon }^{2}+{d}^{2}},{t}_{K^{\prime} }^{1}-\sqrt{{\varepsilon }^{2}+{d}^{2}})$ or $\delta \in ({t}_{K}^{1}+\sqrt{{\varepsilon }^{2}+{d}^{2}},\,{t}_{K^{\prime} }^{1}+\sqrt{{\varepsilon }^{2}+{d}^{2}})$. The ground state is characterized by nonzero Chern number *C* = ±1 if *δ* lies in one nontrivial interval and *C* = ±2 if it is within the overlap of them. Thus we expect one pair or two pairs of edge states in the two topological nontrivial phases.

**Figure 5:**
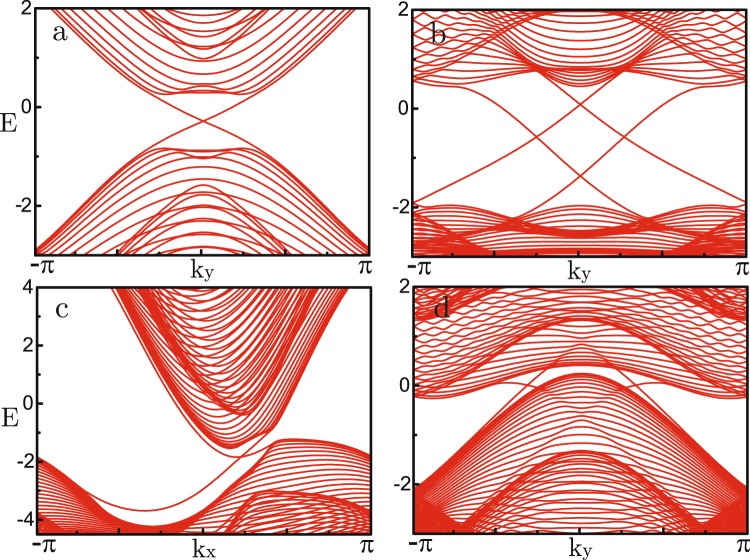
Energy bands for 1D ribbons in the topological nontrivial phases. All figures are parameterized with ${t}_{a}=1,{t}_{b}=0.6,\varepsilon =0.6,\lambda =2.0,d=0.8$. Detuning $\delta =\mathrm{3.5,}\,0$ for one pair (**a**,**c**) and two pair (**b**,**d**) of edge states, whereas magnetic flux $\varphi =2\pi \mathrm{/3,}\,\pi \mathrm{/2}$ for topological insulators (**a**,**b**) and semimetals (**c**,**d**).

The energy spectra of 1D ribbons with armchair and zigzag edges are shown in Fig. 5. As the three-band model, symmetry $\hat{H}({k}_{x},{k}_{y})={\hat{H}}^{\ast }({k}_{x},-\,{k}_{y})$ is also presented if ${\varphi }_{2}={\varphi }_{3}$, resulting in symmetric spectra for the armchair one. We still consider the symmetric cases ${\varphi }_{2}={\varphi }_{3}=\varphi$,${\varphi }_{1}=-\,2\varphi$, with parameters set as ${t}_{a}=\mathrm{1,}\,{t}_{b}=\mathrm{0.6,}$ $\lambda =\mathrm{2.0,}\,\varepsilon =\mathrm{0.6,}\,d=0.8$. For the isotropic case *ϕ*_*i*_ = 2*π*/3, the two nontrivial intervals are (−4.9, 2.3) and (−2.9, 4.3). We find topological insulator phases characterized by one or two pair of edge states when resonance detuning $\delta =3.5$ or $\delta =0$ (Fig. 5a and b). For the other symmetric case *ϕ* = *π*/2, the two nontrivial intervals are (−3.97, 1.57) and (−1.97, 3.57). The two topological insulator phases become semimetal phases at the same detuning (Fig. 5c and d). From the numerical results, we clearly see that the number of edge states accord with our analytical expectation. Like the previous three-band model, Peierls phases *ϕ*_*i*_ and detuning *δ* play the key role to realize topological nontrivial phases.

## Discussion

In conclusion, we have proposed and studied two generalized Haldane models on laser-coupling optical lattice. Laser-assisted NN tunnelings generate artificial staggered magnetic flux, facilitating the realization of topological nontrivial band structures. For the three-band model, we show a simple rule for computing Chern numbers, which are obtained from the values of energy *E*_*n*_(*k*) at the two Dirac points. We also show the analytical phase diagrams of two symmetric cases, verified by numerical calculations of energy spectra. For the four-band model, the Peierls phase depend on the sublattice indices instead of internal states. Different topological nontrivial phases characterized by one pair or two pairs of edge states, can be easily transformed to each other by changing the resonance detuning *δ* and artificial magnetic flux *ϕ*. The effects of laser coupling on topological band structures are discussed. Compared with Haldane model, topological properties of our models are much richer, featuring high Chern number and easy control of topological insulator-semimetal phase transitions. For the staggered artificial magnetic flux, our models can be viewed as two new family members for generalizing Haldane model on optical lattice.

## Methods

Here we show simple rules for computing Chern numbers of our models. For the three-band model, the Bloch wave functions of *H*_*k*_ in equation (4) take the form

12 $${\psi }_{n,k}={({\delta }_{nb}^{k}{\delta }_{nc}^{k}-|{\lambda }_{b}^{k}{|}^{2},{\lambda }_{a}^{k}{\delta }_{nb}^{k},{\lambda }_{a}^{k}{\lambda }_{b}^{k})}^{T},$$

where ${\delta }_{nb(c)}^{k}={E}_{n}(k)-{\xi }_{b(c)}^{k}$ with *E*_*n*_(*k*) the energy of n-th band. If wave function is analytic in the whole Brillouin zone (BZ), the contour integral in equation (6) is zero because of the periodicity of BZ. Nonzero integral comes from singular points, or rather, the two Dirac points $K(K^{\prime} )=(\pm \frac{4\pi }{3\sqrt{3}},0)$. Actually near the Dirac points, ${\lambda }_{a(b)}^{k}$ and ${\delta }_{nn}^{k}$ approach zero, resulting in a vanishing wave function. Here one can use $n=a,b,c$ replacing $n=\mathrm{1,}\,\mathrm{2,}\,3$ to identify energy bands. Expanding them to the second order, we obtain the normalized wave functions

13 $$\begin{array}{rcl}{\psi }_{a,q} & = & {\psi }_{a,q^{\prime} }={(1,0,0)}^{T},\\ {\psi }_{b,q} & = & {(0,0,{e}^{-i2{\theta }_{q}})}^{T},\,{\psi }_{b,q^{\prime} }={(0,0,{e}^{i2{\theta }_{q}})}^{T},\\ {\psi }_{c,q} & = & {(0,{e}^{-i{\theta }_{q}},0)}^{T},\,{\psi }_{c,q^{\prime} }={(0,{e}^{i{\theta }_{q}},0)}^{T}.\end{array}$$

where ${\psi }_{n,q}={\psi }_{n,K+q}$ and ${\psi }_{n,q^{\prime} }={\psi }_{n,K^{\prime} +q}$ with *θ*_*q*_ the axial angle of small momentum *q*. Contribution to integral at the two Dirac points is $\gamma =-\,{\int }_{0}^{2\pi }d\theta \langle \psi |i{\partial }_{\theta }|\psi \rangle =0,\mp \,4\pi ,\mp \,2\pi$, respectively. As a consequence, the Chern number of each energy band is $C=\frac{1}{2\pi }({\gamma }_{K}+{\gamma }_{K^{\prime} })$. So we conclude that the energies at the two Dirac points determine the Chern number of each band. It’s worth noting that the Chern numbers are always zero if ${\varphi }_{i}=0$ in equation (5), because the energy dispersion relations for each band are unchanged at the two Dirac points. For the four-band model, Chern numbers can be computed in a similar manner, which is summarized as the relation of detuning *δ* with the two topological nontrivial intervals $({t}_{K}^{1}\pm \sqrt{{\varepsilon }^{2}+{d}^{2}},\,{t}_{K^{\prime} }^{1}\pm \sqrt{{\varepsilon }^{2}+{d}^{2}})$.

The numerical simulations were done with the tight-binding Hamiltonian (1) and (8) on 1D ribbons of width 25 and 30, respectively. The symmetric and non-symmetric energy spectra correspond to different boundary conditions of armchair and zigzag edges, respectively.
